# Epidural abscess caused by *Streptococcus milleri *in a pregnant woman

**DOI:** 10.1186/1471-2334-5-100

**Published:** 2005-11-03

**Authors:** Russell Lampen, Gonzalo Bearman

**Affiliations:** 1 Division of Infectious Diseases, Department of Internal Medicine, Virginia Commonwealth University Medical Center, Richmond VA, USA; 2 Quality Health Care, Department of Internal Medicine, Virginia Commonwealth University Medical Center, Richmond VA, USA

## Abstract

**Background:**

Bacteria in the *Streptococcus milleri *group (*S. anginosus, S. constellatus, and S. intermedius*) are associated with bacteremia and abscess formation. While most reports of *Streptococcus milleri *group (SMG) infection occur in patients with underlying medical conditions, SMG infections during pregnancy have been documented. However, SMG infections in pregnant women are associated with either neonatal or maternal puerperal sepsis. Albeit rare, *S. milleri *spinal-epidural abscess in pregnancy has been reported, always as a complication of spinal-epidural anesthesia. We report a case of spinal-epidural abscess caused by SMG in a young, pregnant woman without an antecedent history of spinal epidural anesthesia and without any underlying risk factors for invasive streptococcal disease.

**Case presentation:**

A 25 year old pregnant woman developed neurological symptoms consistent with spinal cord compression at 20 weeks gestation. She underwent emergency laminectomy for decompression and was treated with ceftriaxone 2 gm IV daily for 28 days. She was ambulatory at the time of discharge from the inpatient rehabilitation unit with residual lower extremity weakness.

**Conclusion:**

To our knowledge, this is the first reported case of a *Streptococcus milleri *epidural abscess in a healthy, pregnant woman with no history of epidural anesthesia or invasive procedures. This report adds to the body of literature on SMG invasive infections. Treatment of SMG spinal-epidural abscess with neurologic manifestations should include prompt and aggressive surgical decompression coupled with targeted anti-infective therapy.

## Background

*Streptococcus milleri *group (SMG) bacteria are associated with localized abscess formation, most notably in the liver and brain [1]. SMG bacteremia frequently results from occult abscesses, endocarditis, or an underlying gastro-intestinal malignancy [2]. Spinal epidural abscess with SMG are rare, but have been described in individuals with previous epidural anesthesia or malignancy. We report a spinal epidural abscess in a healthy, pregnant woman.

## Case presentation

A 25 year old woman (G2P1) at 20 weeks gestation was in her usual state of good health until 2 weeks prior to hospital presentation. She developed progressive inter-scapular pain followed by lower extremity paresthesias. On the day of admission she noted severe weakness in both legs which developed over the course of 3–4 hours, resulting in the inability to ambulate. She denied fever, rigors, nausea, emesis, or diarrhea prior to admission. She also reported urinary hesitancy for 2–3 days prior to admission, with loss of bladder control on the day of admission. There was no loss of bowel control. At the time of hospital presentation, she was awake, alert, oriented, and not in acute cardiopulmonary distress. The vital signs were stable with a blood pressure of 134/82 mmHg, temperature 97.8°F, pulse 76/minute, and a respiratory rate of 18/minute. She was unable to stand or ambulate. Physical exam revealed ascending sensory deficits to the T4 region below her breasts. Ankle reflexes were absent bilaterally. Patellar reflexes were 1/4 bilaterally and Babinski reflexes were up-going bilaterally. Initial laboratory data revealed a leukocytosis of 18,600/μL (normal 3,700–9,700/μL) hemoglobin of 10.4 gm/dL (normal 12.0–15,0 gm/dl), and electrolytes were within normal limits. Blood cultures were not obtained at time of admission.

**Figure 1:**
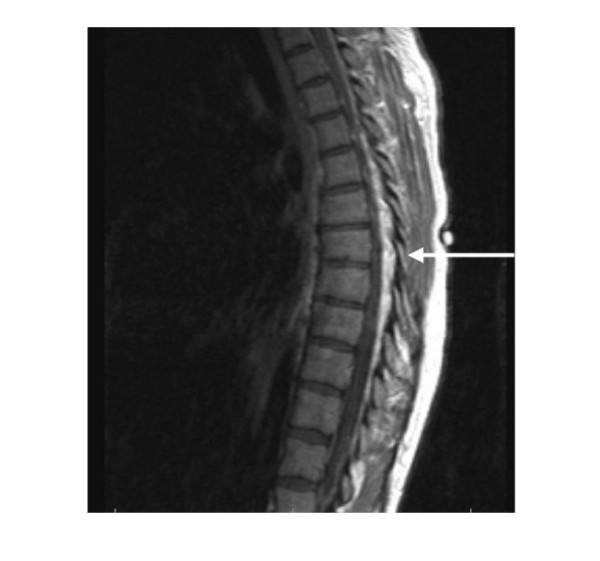
multi-planar, multi-sequence MRI imaging of the thoracic spine. Pre and post contrast sagittal and axial T1, and turbo T2 sequences acquired. An irregular masslike density predominantly T1 and T2 hyperintense with a serpiginous contour is demonstrated in epidural location, as a mass surrounding the thoracic cord. The lesion extends from the T1 level distally through the lower thoracic spine.

An MRI of the thoracic spine, performed at the time of presentation, revealed an irregular mass-like density predominantly at the T1 and T2 levels, with a hyper-intense and serpiginous contour demonstrated in the epidural space (Figure 1). Spinal cord volume loss due to compression was noted in the upper thoracic segments. Owing to the spinal cord compression and associated neurologic symptoms, an emergent laminectomy was performed. Operative tissue samples were sent for pathology and microbiologic analysis. Histopathology revealed nonspecific chronic, active inflammation. Gram stain of the specimens was negative. Operative wound cultures grew *Streptococcus milleri *on the 3^rd ^post-operative day. Two sets (2 anaerobic and 2 aerobic collection bottles) of blood cultures obtained upon receiving the results of the wound cultures and prior to initiating antibiotic therapy were negative. Vaginal cultures were not obtained either prior or post-operatively to determine if the patient was colonized with SMG.

The patient was treated with ceftriaxone 2 gm IV daily for 28 days. An MRI of the abdomen and pelvis revealed no focal fluid collections or abscesses. A transesophageal echocardiogram revealed no valvular pathology. She was discharged to home after 21 days and was able to ambulate with the assistance of a walker. At the time of hospital discharge, partial improvement in the sensory deficit was noted. Six weeks after initial therapy, residual sensory and motor deficits persisted, requiring the patient to ambulate with the aid of a walker. The patient gave birth to a healthy baby boy by vaginal delivery. Ten months after the initial presentation, the patient was ambulating without a walker and had near complete recovery of both the motor and sensory deficits.

## Discussion

Spinal epidural abscess is a rare condition that occurs even more infrequently in pregnancy. Hunter and colleagues described a case of *S. aureus *spinal epidural abscess secondary to posterior vertebral osteomyelitis in a previously healthy, 27 year old pregnant woman [3]. Surgical decompression and antibiotic therapy with intravenous methicillin and gentamicin resulted in prompt recovery and improvement of a mild neurologic deficit.

In another case report, a 22 year old woman presented with infrascapular back pain, paresthesias of the thighs, difficulty on ambulation, and fever 6 days post partum [4]. The delivery had been unremarkable and the patient had not received epidural analgesia. Myelography revealed a complete block at the level of the 4^th ^thoracic vertebrae. An emergent, upper thoracic laminectomy was performed. The causative agent was penicillin *sensitive S. aureus*. Despite surgical decompression and systemic antibiotics, the neurologic deficits failed to reverse.

While *S. aureus *has been implicated in spontaneous epidural abscess during pregnancy, *S. milleri *group bacterial epidural abscess has to date only been described following epidural anesthesia [5,6]. The first case described by Gelfand et al [5] involved a 31 year old woman who had a lumbar epidural catheter placed for anesthesia during vaginal delivery. She returned to the hospital 11 days later with signs and symptoms of spinal compression. A laminectomy was performed and she received a 21 day course of appropriate antibiotics.

A second case recently reported by Schroeder and colleagues[6] described an epidural abscess caused by SMG that occurred following the placement of an epidural catheter for anesthesia in an uncomplicated full term vaginal delivery. The catheter was in place for 6 hours intrapartum and was removed promptly following delivery. The patient developed signs of spinal cord compression on post-partum day 5 and was found to have an epidural abscess by MRI. Laminectomy was performed and the patient was treated with ceftriaxone and clindamycin for 4 weeks.

Although rare, *S. milleri *has been previously implicated in post-partum spinal-epidural abscesses. However, prior cases were associated with the placement of an invasive, epidural catheter. This likely served as the portal of entry into the spinal-epidural space.

*Streptococcus milleri *Group (SMG) organisms were previously known as *Streptococcus anginosus *or *Streptococcus milleri-anginosus *group. They are gram-positive cocci distinguished by their microaerophilic growth requirements, their formation of colonies <0.5 mm in diameter, and by the presence of a distinct caramel-like odor when cultured [1]. SMG organisms are commensals of the oral cavity and of the gastrointestinal tract. SMG organisms are notorious causes of pyogenic, invasive infections, and have been found in head and neck abscesses, bacteremia with endocarditis, liver abscess, thoracic empyema, brain abscess, and spinal epidural abscess [1]. Although unusual, infective endocarditis by SMG organisms has been reported [1]. Patients with underlying medical conditions, such as cirrhosis, diabetes mellitus, and malignancies, are predisposed to invasive infections with *Streptococcus milleri *[2].

Colonization of the vagina has also been noted [1]. In a revew of 214 fetal necropsies from second term spontaneous abortions, MacGowen found 40 cases of chorioamnionitis or intrauterine pneumonia; SMG were implicated in 5 of these infections. In two of the five cases, maternal vaginal swabs taken 24 hours prior to delivery showed profuse growth of *S. milleri *[7]. In a similar study that reviewed clinical specimens of neonates suspected of neonatal infection, S. milleri was found in 7.9% of the 2,510 neonates examined [8]. While these studies demonstrate the pathogenic potential and extent of vaginal colonization with *S. milleri *in prenatal and neonal infections, it remains unknown if pregnancy increases vaginal colonization.

Spinal epidural abscesses in the non-pregnant population due to *S. milleri *are also uncommon [5,9-11]. All reported cases involve patients with either a malignancy, a recent epidural catheter, or spinal surgery [1,3,5,6] Our case was unusual as this patient was a healthy, young woman without history of prior surgical intervention, malignancy, or chronic illness. To our knowledge, this is the first reported case of a *Streptococcus milleri *epidural abscess in a healthy, pregnant woman. Previous reports of pregnancy related SMG infections are associated with neonatal sepsis or maternal puerperal sepsis but not spinal epidural abscess [3].

The etiology of the patient's spinal epidural abscess is unclear. She may have been transiently bacteremic with *S. milleri *from either an oral, gastrointestinal, or vaginal source with consequent seeding of the central nervous system. Apart from a pelvic examination performed early in the first trimester of pregnancy, no other invasive procedures were reported.

Our decision to treat for 28 days with ceftriaxone was consistent with prior reports of successful treatment for SMG spinal-epidural abscess in non-pregnant women [1]. Emergent surgical decompression and drainage is needed if there is evidence of neurological impairment [12]. For spinal-epidural abscesses without motor or sensory deficits, antibiotic therapy alone may be considered [12]. Significant neurological improvement is unlikely when surgical decompression is delayed greater than 24 hours.

## Conclusion

We report a case of spinal-epidural abscess caused by SMG in a young, pregnant woman. The patient had no underlying medical conditions, including cirrhosis, diabetes mellitus, and malignancies, and had not undergone any prior invasive procedures. As such, no risk factors for invasive disease with SMG were identified.

While most reports of SMG infection occur in patients with underlying medical conditions, SMG infections during pregnancy have also been documented. However, reports of SMG infections in pregnancy have typically been associated with either neonatal sepsis or maternal puerperal sepsis, rather than spinal epidural abscess. This case report adds to the literature on pregnancy related SMG infections.

Despite the absence of comorbidities in this case, the degree and rapidity of clinical deterioration was noteworthy and manifested as spinal cord volume loss in the upper thoracic segments with concurrent sensory and motor deficits of the bilateral lower extremities. Given the potential severity of SMG spinal-epidural infections, prompt surgical laminectomy and intravenous antibiotic therapy is of paramount importance.

## Competing interests

The author(s) declare that they have no competing interests.

## Authors' contributions

Both RL and GB were involved in the care of the patient and in the writing of the entire manuscript. Both authors read and approved the final manuscript.

## Pre-publication history

The pre-publication history for this paper can be accessed here:


